# Cerebrovascular Disease and Statins

**DOI:** 10.3389/fcvm.2021.778740

**Published:** 2021-12-02

**Authors:** Luis M. Beltrán Romero, Antonio J. Vallejo-Vaz, Ovidio Muñiz Grijalvo

**Affiliations:** ^1^Internal Medicine, Virgen del Rocío University Hospital, Seville, Spain; ^2^Clinical Epidemiology and Vascular Risk, Instituto de Biomedicina de Sevilla, IBiS/Hospital Universitario Virgen del Rocío/Universidad de Sevilla/CSIC, Seville, Spain; ^3^Department of Medicine, Faculty of Medicine, University of Seville, Seville, Spain

**Keywords:** atherosclerosis, cerebrovascular disease, LDL-cholesterol, statins, stroke

## Abstract

Elevated low-density lipoprotein-cholesterol (LDL-C) is a causal factor for the development of atherosclerotic cardiovascular disease (ASCVD); accordingly, LDL-C lowering is associated with a decreased risk of progression of atherosclerotic plaques and development of complications. Currently, statins play a central role in any ASCVD management and prevention strategies, in relation to their lipid-lowering action and potentially to pleiotropic effects. After coronary artery disease, stroke is the most frequent cause of ASCVD mortality and the leading cause of acquired disability, a major public health problem. There is often a tendency to aggregate all types of stroke (atherothrombotic, cardioembolic, and haemorrhagic), which have, however, different causes and pathophysiology, what may lead to bias when interpreting the results of the studies. Survivors of a first atherothrombotic ischemic stroke are at high risk for coronary events, recurrent stroke, and vascular death. Although epidemiological studies show a weak relationship between cholesterol levels and cerebrovascular disease as a whole compared with other ASCVD types, statin intervention studies have demonstrated a decrease in the risk of stroke in patients with atherosclerosis of other territories and a decrease in all cardiovascular events in patients who have had a stroke. The Stroke Prevention by Aggressive Reduction in Cholesterol Levels (SPARCL) trial demonstrated the benefit of high doses of atorvastatin in the secondary prevention of ischemic stroke. In this review, we discuss the evidence, use and recommendations of statins in the primary and secondary prevention of stroke, and their role in other scenarios such as the acute phase of ischemic stroke, cerebral hemorrhage, cardioembolic stroke, small vessel disease, and cognitive impairment.

## Introduction

Stroke represents a major public health issue, being a leading cause of mortality and of acquired disability in the general population. For instance, stroke is consistently ranking among the first leading diseases in the Global Burden of Diseases reports, specifically the third leading disease in the overall population in year 2019 and second after ischemic heart diseases in the population aged 50 years and over (1). According to data from the Heart Disease and Stroke Statistics 2021 referring to analysis using data from the Global Burden of Diseases, 87% of stroke risk could be attributable to modifiable risk factors, including hyperlipidemia (2), thus, suggesting that most cerebrovascular events could be potentially prevented with timely and effective interventions.

It is well-recognized that low-density lipoprotein cholesterol (LDL-C) is a causal risk factor for the development of atherosclerosis, related to the magnitude of the elevation of LDL-C levels and for how long they remain high (cumulative exposure over time) (3). Lowering LDL-C levels is, therefore, a key intervention to reduce vascular risk, with statins being recommended by clinical guidelines as the first choice drug for LDL-C lowering in patients at increased risk of atherosclerotic cardiovascular disease (4, 5), based on a well-established body of evidence for the benefit and safety of statins to reduce future cardiovascular risk (3–5). As such, statins play a central role in any atherosclerotic cardiovascular disease management and prevention strategies, in relation to their lipid-lowering action but also potentially to a number of other additional beneficial properties (“pleiotropic” effects) beyond their lipid-lowering effect (6). However, while these potential “pleiotropic” effects of statins have been debated extensively, the latest evidence suggests that the beneficial effects of statins in atherosclerotic complications would be mostly the result of their cholesterol-lowering effect (3); this is reinforced by the stroke preventive effects of other non-statin lipid-lowering therapies (such as PCSK9 inhibitors or ezetimibe) and some recent mendelian randomization study, particularly for large artery atherosclerotic stroke (7, 8).

However, the etiology and mechanisms of stroke are heterogeneous and consequently the role of high LDL-C (and so of lipid-lowering interventions) may be variable depending on the type and pathogenesis of stroke (4). While the relationship between high LDL-C and cerebral atherosclerosis and atherothrombotic events are better established, this relationship is uncertain when the stroke results from a different etiology/mechanism, such as cardiac embolisms (e.g., related to atrial fibrillation), small-vessel cardiovascular disease, or intracerebral hemorrhage (4). Consequently, the role and effect of statins in the prevention of cerebrovascular diseases may differ depending on the type of stroke. Additionally, the evidence for atherosclerotic-related strokes may vary with respect to that of atherosclerotic disease in other vascular beds, mostly with coronary artery disease. In the present article we briefly review the role of statins in the primary and secondary prevention of stroke, overall and in certain cerebrovascular disease conditions.

## Statins and Primary Prevention of Stroke

There are no clinical trials specifically designed to assess the role of lipid-lowering therapy in the primary prevention of stroke. Instead, primary prevention studies use to include this outcome as part of combined endpoints together with other events (composite endpoint made of different cardiovascular events), or it is a secondary endpoint with the study design not specifically powered to address this outcome (i.e., reduced statistical power), or it is explored in *post-hoc* analyses. Therefore, while most primary prevention trials with statins focus on coronary artery disease or composite endpoints, conclusions on stroke are rather limited (9). This lack of specific clinical trials in primary prevention is probably driven, at least partly, from the observation of a weak association between hypercholesterolemia and cerebrovascular disease shown by large classical observational studies (which in turn is further limited by the fact that the evidence resulting from observational studies is weaker as compared to randomized clinical trials with hard clinical end-points). This is further compounded by the varied mechanisms responsible for stroke as opposed to the atherosclerotic origin of the vast majority of events in ischemic heart disease, and the paucity of clinical trials differentiating the different subtypes of stroke, i.e., ischemic (thrombotic, embolic…) and hemorrhagic; thus, not accounting for their differences in etiology or pathogenesis. Furthermore, a significant proportion of strokes remained unclassified, and they frequently occur in patients with other concurrent potential causes of stroke (e.g., hypertension, atrial fibrillation…). All these factors together make it complicated to extract clearer conclusions.

There are indirect or secondary data from several primary prevention studies with statins, including ALLHAT-LLT (Antihypertensive and Lipid-Lowering treatment to prevent Heart Attack, Lipid-Lowering Therapy) (10) and WOSCOPS (West of Scotland COronary Prevention Study) (11) clinical trials, both with pravastatin, and the ASCOT-LLA (Anglo-Scandinavian Cardiac Outcomes Trial, Lipid-Lowering Arm) (12) and CARDS (Collaborative Atorvastatin Diabetes Study) (13) trials with atorvastatin. Although there were differences in study design, *post-hoc* analyses of the studies with pravastatin showed a reduction in the relative risk of suffering a first stroke of 9–11%, which was not statistically significant. However, a more intensive lipid-lowering strategy with a high-potency statin (atorvastatin), as used in ASCOT-LLA and CARDS (in the diabetic population), resulted in a greater reduction of LDL-C levels, and it translated into a significant reduction in the relative risk of cerebrovascular events in primary prevention of 27–48%.

To try to overcome some limitations from individual studies (e.g., reduced statistical power), several meta-analyses have been conducted, attempting to elucidate the effect of statins on the risk of a first stroke in the primary prevention of this condition. Briel et al. (9) found a reduction in the relative risk of cerebrovascular events of 23% in patients treated with statins vs. placebo, with this reduction slightly rising to 25% if the patients had a previous coronary artery disease. The CTT (Cholesterol Treatment Trialists' Collaborators) meta-analysis (14) including 90,056 patients in primary prevention found a 17% reduction in the relative risk of any type of stroke for each 1 mmol/L (39 mg/dL) decrease in LDL-C; this means a reduction of 5 and 8 cerebrovascular events per 1,000 participants without and with a previous history of coronary heart disease, respectively, among patients treated with statins vs. placebo during an average follow-up of 5 years. In the meta-analysis by Amarenco et al. (15), including a total of 165,792 patients at high risk of stroke, it was reported an 18% reduction in all types of stroke in patients treated with statins (95% confidence interval [CI] 13–23%, *p* < 0001) with no increase in hemorrhagic strokes and a non-significant reduction in fatal strokes of 13%.

## Statins and Secondary Prevention of Stroke

Most trials with statins in the secondary prevention of stroke share similar limitations with primary prevention studies, as described above; additionally, they include no or few patients with previous stroke at baseline, with the notable exceptions of the HPS (Heart Protection Study) (16), SPARCL (Stroke Prevention by Aggressive Reduction in Cholesterol Levels) (17) and the more recent TST (Treat Stroke to Target) (18, 19) trials.

The HPS was the first study to evaluate the benefit of statins, specifically simvastatin, in the secondary prevention of stroke in patients with a previous history of cerebrovascular disease (16). This study included 20,536 patients with high cardiovascular risk, of whom 3,280 had a previous stroke or transient ischemic attack, who were randomized to receive simvastatin 40 mg or placebo. After an average of 5-year follow-up, a significant decrease in major cardiovascular events was observed (more evident in the subgroup of patients without previous coronary heart disease); however, it was not found a significant reduction in the risk of recurrent stroke specifically (10.4% in the treatment group vs. 10.5% in the placebo group). One of the suggested reasons that might explain this lack of effect was the late inclusion of patients after their first stroke event (4.3 years), when the risk of recurrence of stroke is lower. Despite the latter point, the benefits the in total reduction of major cardiovascular events and mortality in patients with prior stroke led the USA Food and Drug Administration (FDA) to approve the indication of statins in patients with stroke (20).

The SPARCL study was designed to specifically elucidate the role of statins in the secondary prevention of cerebrovascular disease (17). This trial included 4,731 patients with a history of a recent (6–17 months prior to study randomization) stroke or transient ischemic attack (67% corresponded to ischemic stroke, 31% to transient ischemic attack, and 2% to hemorrhagic stroke) without prior history of clinical coronary heart disease. Patients were randomly assigned to receive atorvastatin 80 mg or placebo in a 1:1 ratio, and were followed-up for 4.9 years. The primary endpoint was the time to a first fatal or non-fatal stroke. In the treatment group, a 16% reduction in the relative risk of recurrent stroke was observed, with this percentage rising to 43% for the reduction of fatal strokes specifically (*p* = 0.03). Similarly, a relative decrease in the risk of coronary episodes of 35% was observed in patients receiving atorvastatin vs. placebo. It should also be noted that the incidence of hemorrhagic stroke was higher in the group treated with atorvastatin: 55 cases, compared to 33 cases in the placebo group, which represents an increase in the relative risk of 66%; this point raised some controversy over the results of this study. This aspect is more extensively discussed in a later section of this article. Another controversial point of SPARCL is that, from the analyses of the LDL-C levels during the follow-up, it is inferred that up to 25% of patients assigned to placebo received statin treatment at some point during the study follow-up, which, in addition to a protocol deviation bias, could have led to an underestimation of both the atorvastatin-related reduction in the risk of recurrence of ischemic stroke and the potential increased risk of hemorrhagic stroke. In a subsequent sub-analysis in which patients were categorized according to their level of LDL-C reduction (21) it was found, however, that among patients in whom their LDL-C levels experienced a reduction >50%, no increase in the risk of cerebral bleeding was detected whereas the benefit in terms of recurrence of any subtype of stroke and of major coronary events was of 31% and 37% relative risk reduction, respectively. Based on this study, the use of statins in patients with stroke caused by atherosclerosis was generalized in routine clinical practice worldwide (4, 5, 22).

Finally, the Treat Stroke to Target trial was designed to assess the optimal target level for LDL-C to reduce overall cardiovascular events among patients with a recent ischemic stroke (in the prior 3 months) or transient ischemic attack (in the prior 15 days) in patients with evidence of atherosclerosis (18). Two thousand eight hundred sixty patients were randomly assigned to a target LDL-C level of <70 mg/dL or to a target range of 90–110 mg/dL and treated with statins, ezetimibe or both. After a median follow-up of 3.5 years, patients who were assigned to the lower-target group had a lower risk of major cardiovascular events compared to those in the target range of 90–110 mg/dL (8.5 vs. 10.9% events, respectively; adjusted Hazard Ratio [HR] 0.78 [95% CI 0.61–0.98]). While there was a numerically higher number of intracranial hemorrhages in the lower-target group (1.3, vs. 0.9% in the higher-target group) the between-group comparison was not significant (HR 1.38; 95% CI: 0.68–2.82) (18). The trial was, however, terminated early for administrative reasons and the expected number of primary end-point events was not reached (277 of the anticipated 385 end-point events had occurred). However, a later anlaysis of the subgroup of patients (French cohort, *n* = 1,073) of the trial that had a longer follow-up (median 5.3 years) resulted in concordant results for both a reduction of risk of major cardiovascular events (9.6% and 12.9% in the lower- and higher-target groups, respectively; HR: 0.74 [95% CI: 0.57–0.94]) and a no significant difference in the occurrence of intracranial hemorrhages (HR: 1.17, 95% CI: 0.53–2.62) (19).

The results from the TST trial support the current guideline recommendations of intensive lipid-lowering treatment to reduce the LDL-C levels in patients with established atherosclerotic cardiovascular disease, including stroke (4, 5). Although LDL-C therapeutic goals can be achieved with statins in monotherapy, a significant proportion of patients at high and very high risk with high LDL-C will still require additional drug therapy. In this clinical scenario, in which the therapeutic goal is not achieved despite statin therapy at the maximum tolerated dose, the combination with ezetimibe and/or PCSK9 inhibitors may be needed (4, 5). At this respect, the results from IMPROVE-IT (Improved Reduction of Outcomes: Vytorin Efficacy International Trial) (23, 24) trial of simvastatin in combination with ezetimibe, vs. statin alone, in patients stabilized after an acute coronary syndrome, showed a 21% risk reduction of ischemic stroke (HR: 0.79; 95% CI: 0.67–0.94) with the combination therapy after a 6-year median follow-up; this risk reduction was larger among patients with a history of a prior stroke (HR: 0.52; 95% CI, 0.31–0.86) (24). Results from randomized controlled trials of PCSK9 inhibitors added on top of statins, including FOURIER (Further Cardiovascular Outcomes Research with PCSK9 Inhibition in Subjects with Elevated Risk) with evolocumab (25, 26), ODYSSEY OUTCOMES (Evaluation of Cardiovascular Outcomes After an Acute Coronary Syndrome During Treatment With Alirocumab) with alirocumab (27, 28) and other trials, have reported results along the same lines in secondary prevention patients (29); in particular, a meta-analysis of 39 randomized clinical trials including over 66,000 individuals observed a risk reduction of ~22% (*p* < 0.001) with PCSK9 inhibitors vs. control (29).

The effects of statins on other types of ischemic stroke (e.g., embolic, small vessel disease) has not been adequately tested in randomized clinical trials, and observational epidemiological studies or imaging studies may not provide a robust evidence to support clinical indications. However, given the fact that in most cases these patients may be characterized as having global atherosclerotic cardiovascular risk (presence of cardiovascular risk factors, subclinical or established atherosclerosis), statin therapy may be indicated for global atherosclerotic cardiovascular disease prevention, and thus may merit statin therapy regardless of stroke considerations (4, 5). Specific scenarios and some subtype of strokes are described in more detail in the next sections.

## Specific Scenarios

### Acute Ischemic Stroke

As discussed above, there is evidence supporting the use of statins after an ischemic stroke or a transient ischemic attack to prevent further events and, consequently, the main clinical practice guidelines recommend their use in this situation (4, 5). However, there is no such a robust evidence to support the routine use of statins in the acute phase of stroke (first 2 weeks). Based on a lower level of evidence, clinical practice guidelines generally indicate that in-hospital continuation or initiation of statin therapy after an acute ischemic stroke is reasonable (30, 31).

There is a limited number of published randomized clinical trials examining the role of early statin use in acute ischemic stroke patients. The FASTER (Fast Assessment of Stroke and Transient Ischemic Attack to Prevent Early Recurrence) study evaluated the effect of simvastatin 40 mg vs. placebo in patients with a transient ischemic attack or minor stroke within the previous 24 h (32). The trial was terminated early due to a slow recruitment; no significant differences in recurrent stroke or safety outcomes were found. However, FASTER was underpowered and it used a moderate-intensity statin, both factors that could have impacted the results. Another randomized trial, ASSORT (Administration of Statin on Acute Ischemic Stroke Patient), found no difference in the functional performance at 90-day when statins were initiated within 24 h or in the 7th day (33). On the other hand, a meta-analysis mainly including observational studies found that in-hospital statin use was associated with a better functional outcome and a trend toward a lower short-term mortality (34); moreover, importantly, the withdrawal of pre-stroke statin therapy during an hospitalization for an acute ischemic stroke was associated with poorer functional outcomes. In patients who were treated with thrombolysis the beneficial effects on functional outcomes were also observed despite an increased risk of hemorrhagic transformation.

Another additional factor to consider is the suggestion that the initiation of statin therapy during the hospitalization for an acute ischemic stroke event could have medium to long term benefits derived from a better adherence of patients to the statin therapy (35). Finally, beyond cardiovascular outcomes, in-hospital statin therapy may reduce the risk of seizures after an acute ischemic stroke event if initiated promptly (36).

### Cerebral Small Vessel Disease and Cognitive Impairment

Cerebral small vessel disease (CSVD) is a pathologic process commonly associated with atherosclerosis and cardiovascular risk factors such as hypertension, hyperlipidemia, and aging (37). 25–30% of strokes are attributable to CSVD and it increases the odds of recurrent stroke by over 2-fold (38, 39). Moreover, CSVD is also strongly associated to cognitive decline and dementia, contributing to 45% of dementia cases (40) and to functional decline in the elderly (41).

There is some evidence supporting the protective effects of statins on progression of CSVD and its consequences. Most of this evidence has been related to the progression of white matter hyperintensities (WMH) and cognitive decline as markers of CSVD. Initial studies on statins and CSVD showed a potential, but not yet confirmed, benefit of these drugs on CSVD. For instance, the PROSPER (Prospective Study of Pravastatin in Elderly at Risk) trial with pravastatin showed a trend toward a lower number of new infarcts in the treatment arm but failed to demonstrate an effect on the progression of WMH volumes (42). The Cardiovascular Health Study reported that statin use was not associated with a change in visually rated WMH severity over 5 years despite a reduced rate of cognitive decline in the statin-treated group (43).

Most recent studies using newer MRI protocols and quantitative evaluation of WMH have shown more concordant results. In a cohort of older adults living in the community (non-institutionalized), statins were associated to a more favorable disease progression in the sub-group of lowest cognitive performance (44). Xiong et al. found that in patients with CSVD statin therapy was associated with a decreased risk of stroke, progression of WHM and cognitive decline (45). In the same line, a more recent randomized clinical trial which included 732 hypertensive patients aged 60 years and older followed for a mean of 5 years found that patients allocated to rosuvastatin had a reduced progression of WMH and less cognitive decline compared to placebo (46). Finally, Guo et al., using data of two previous studies (prospective cohort and randomized clinical trial) found that adults 75 years and older taking statins had a lower progression of CSVD without increasing the risk of microbleeds (47).

All these results together, particularly those from more contemporary studies, support the hypothesis that statin therapy may have a protective effect on cognitive decline through a reduced progression of CSVD. There is however controversy about the potential benefit of statin therapy on other cognitive disorders with a different pathophysiology, such as Alzheimer's disease, although most evidence point toward a neutral effect of statins on Alzheimer's disease features (48, 49); some evidence showed a potential, though modest reduction in long-term risk of dementia after a concussion in older adults taking statins (50).

### Intracerebral Hemorrhage

There is a long-lasting and ongoing controversy about the relationship between statin therapy and intracerebral hemorrhage. This is based on the safety results from the randomized clinical trials SPARCL and HPS, which included patients with a prior stroke or transient ischemic attack, and which reported an increased risk of intracerebral hemorrhage among patients allocated to the statin arm compared with control (17, 51). This risk seemed particularly enhanced in patients with prior intracerebral hemorrhage (52). However, subsequent sub-analysis of these trials and later studies including the TST trial (as discussed before) have not confirmed this association (16–19, 21, 53–56). For instance, a meta-analysis of 31 randomized clinical trials of statins did not find any increase in the risk of intracerebral hemorrhage among statin-treated patients, while reporting a reduction in all-cause mortality and all stroke types with statin therapy (53). A large Danish population-based study including 2,728 participants with intracerebral hemorrhage and 52,964 participants with ischemic stroke, found no evidence that statins increase the risk of intracerebral hemorrhage in individuals with prior stroke (54); in fact, the risk of recurrent intracerebral hemorrhage was similar for statin users and non-users among those with prior intracerebral hemorrhage, while this therapy was associated with a decreased risk of intracerebral hemorrhage in those with prior ischemic stroke (54). The same group reported that, in a population without prior history of stroke, those taking statins had lower intracerebral hemorrhage risk compared with those who did not receive statin therapy (55). Another large population-based study in Korean hyperlipidemic patients without history of haemorrhagic stroke, found that statin therapy was associated with a decreased risk of intracerebral hemorrhage and with improvements in ischemic cardiovascular and cerebrovascular outcomes (56). According to some observational studies, the potential link between statin use and intracerebral hemorrhage may be enhanced in patients with a background of cerebral angiopathies such as cerebral amyloid angiopathy or CADASIL (Cerebral Autosomal Dominant Arteriopathy with Sub-cortical Infarcts and Leukoencephalopathy) (57).

In this context, current clinical guidelines state that there are insufficient data to recommend restrictions on statin use in patients with prior stroke but recommend caution in patients with spontaneous intracerebral hemorrhage, specially in the subgroup of patients with cerebral amyloid angiopathy-related lobar intracerebral hemorrhage, in which statin therapy should probably be reserved for compelling indications (very high risk of atherotrombotic events) (30).

### Cardioembolic Stroke

There is scarce evidence of the effect of statin therapy in patients with cardioembolic stroke in the absence of a known indication for statins and, consequently, clinical practice guidelines do not present any specific recommendation at this respect. Because cardioembolic stroke can be linked to atherosclerotic disease (e.g., aortic plaques) and cardiovascular risk factors (e.g., hypertension and left hypertensive cardiomyopathy) statins might also potentially influence the outcomes in these patients. However, no randomized clinical trials addressing specifically this question have been conducted; moreover, randomized clinical trials with statins often exclude patients with cardiac sources of embolism, as occurred in SPARCL (17).

Some evidence from observational studies in patients with cardioembolic or atrial fibrillation-related stroke suggests an association of statin therapy with a lower incidence of vascular events and mortality. A retrospective study by Choi et al. including 535 patients with a first-ever cardioembolic stroke found that statin therapy was independently associated with a reduced mortality (58). In the same line, another study using data from a registry of patients with atrial fibrillation observed that treatment with statins was associated with improved survival and reduced risk of future cardiovascular events (59). Finally, a recent study analyzed data from a large prospective multicenter stroke registry and reported that statin therapy may reduce the risk of major vascular events, vascular death, and all-cause death in patients with acute cardioembolic stroke with no clear indication for statin therapy according to clinical guidelines (60).

## Conclusions

While stroke be may derived from a variety of pathologic processes different from atherosclerosis, there is often a tendency to aggregate all types of stroke (atherothrombotic, cardioembolic, small vessel disease, haemorrhagic…) together, which have, however, different etiologies and pathophysiologic mechanisms, what may lead to bias when interpreting the results of the studies.

Although epidemiological studies showed a weaker relationship between cholesterol levels and cerebrovascular disease as a whole compared with other atherosclerotic cardiovascular disease types, statin intervention trials suggest a beneficial role of statin therapy on cardiovascular, risk reduction, including stroke, particularly in the secondary prevention of stroke from atherosclerotic vascular disease. The SPARCL trial demonstrated the benefit of high doses of atorvastatin in the secondary prevention of ischemic stroke and the TST trial supports intensive lipid-lowering therapy to achieve guideline recommended LDL-C targets of at least <70 mg/dL. The effects of statins are likely mediated by LDL-C reduction, as also supported by the results from trials with other cholesterol-lowering drugs (to which no pleitropic effects similar to statins have been attributable), which have also shown stroke protection in secondary prevention trials in combination with statins. The potential role for statins in the prevention of other different stroke subtypes such as small vessel stroke or cardioembolic stroke, or in cognitive impairment, has not been adequately evaluated; however, since these clinical conditions frequently occur in patients with increased atherosclerotic cardiovascular risk, most patients with these conditions may benefit from statin treatment based on global cardiovascular risk prevention.

Further studies and evidence are required, mostly in the primary prevention of stroke and among those cerebrovascular conditions where the atherosclerotic process is not the main mechanism.

## Author Contributions

OMG conceived the present review. All authors reviewed the literature, contributed to the interpretation of the evidence, wrote the manuscript, and approved its submission for publication.

## Conflict of Interest

LBR reports past participation in investigator-initiated research grants to Hospital Universitario La Paz from MSD; personal fees for consulting/advisory committee from Sanofi-Aventis, Amgen and Bayer; honoraria for lectures from Pfizer, MSD, Novartis, Sanofi-Aventis and Amgen; all outside the submitted work. AV-V reports current or past participation in investigator-initiated research grants to Imperial College London and/or European Atherosclerosis Society from Pfizer, Amgen, MSD, Sanofi-Aventis, Daiichi-Sankyo and Regeneron; personal fees for consulting/advisory committee from Bayer, Regeneron and Radcliffe Cardiology; honoraria for lectures from Amgen, Mylan and Akcea; all outside the submitted work. OMG reports current or past participation in investigator-initiated research grants to Hospital Universitario Virgen del Rocío from MSD, Astra-Zeneca and Sanofi-Aventis; personal fees for consulting/advisory committee from MSD, Ackcea, Sanofi-Aventis, Novartis and Daiichi-Sankyo; honoraria for lectures from MSD, Astra-Zeneca, Sanofi-Aventis, Daiichi-Sankyo, Novartis, Amgen, Ferrer, Esteve, Mylan-Viatris and Pfizer; all outside the submitted work.

## Publisher's Note

All claims expressed in this article are solely those of the authors and do not necessarily represent those of their affiliated organizations, or those of the publisher, the editors and the reviewers. Any product that may be evaluated in this article, or claim that may be made by its manufacturer, is not guaranteed or endorsed by the publisher.
